# Developmental Language Disorder: Wake and Sleep Epileptiform Discharges and Co-morbid Neurodevelopmental Disorders

**DOI:** 10.3390/brainsci10120910

**Published:** 2020-11-26

**Authors:** Olga Dlouha, Iva Prihodova, Jelena Skibova, Sona Nevsimalova

**Affiliations:** 1 Department of Phoniatrics, 1st Faculty of Medicine, Charles University and General Hospital, 12000 Prague, Czech Republic; olga.dlouha@vfn.cz; 2 Department of Neurology and Clinical Sciences, 1st Faculty of Medicine, Charles University and General Hospital, 12000 Prague, Czech Republic; iva.prihodova@lf1.cuni.cz; 3 Unit of Statistics, Institute of Clinical and Experimental Medicine, 14000 Prague, Czech Republic; jesk@ikem.cz

**Keywords:** developmental language disorder, DLD, developmental coordination motor disorder, ADHD, receptive and expressive DLD form, perinatal risk factors, wake EEG, nocturnal EEG, polysomnography, epileptiform discharges, spindle activity, memory and language

## Abstract

Developmental language disorder (DLD) is frequently associated with other developmental diseases and may lead to a handicap through adolescence or adulthood. The aim of our retrospective study was to characterize DLD subgroups, their etiological factors and clinical comorbidities, and the role of epileptiform discharges in wake and sleep recordings. Fifty-five children (42 male, mean age 6.2 ± 1.4 years, range 4–9 years) were included in the present study and underwent phoniatric, psychologic, neurologic, as well as wake and nocturnal electroencephalography (EEG) or polysomnography (PSG) examinations. A receptive form of DLD was determined in 34 children (63.0%), and an expressive form was found in 20 children (37.0%). Poor cooperation in one child did not permit exact classification. DLD children with the receptive form had significantly lower mean phonemic hearing (79.1% ± 10.9) in comparison with those with the expressive form (89.7% ± 6.2, *p* < 0.001). A high amount of perinatal risk factors was found in both groups (50.9%) as well as comorbid developmental diseases. Developmental motor coordination disorder was diagnosed in 33 children (61.1%), and attention deficit or hyperactivity disorder was diagnosed in 39 children (70.9%). Almost one half of DLD children (49.1%) showed abnormalities on the wake EEG; epileptiform discharges were found in 20 children (36.4%). Nocturnal EEG and PSG recordings showed enhanced epileptiform discharges, and they were found in 30 children (55.6%, *p* = 0.01). The wake EEG showed focal discharges predominantly in the temporal or temporo-parieto-occipital regions bilaterally, while in the sleep recordings, focal activity was shifted to the fronto-temporo-central areas (*p* < 0.001). Almost all epileptiform discharges appeared in non-rapid eye movement (NREM) sleep. A close connection was found between DLD and perinatal risk factors, as well as neurodevelopmental disorders. Epileptiform discharges showed an enhancement in nocturnal sleep, and the distribution of focal discharges changed.

## 1. Introduction

Developmental language disorder (DLD), previously termed specific language impairment (SLI), is a common developmental disorder that affects approximately 7% of preschool-aged children [1]. It is characterized by language skills that are substantially below age-appropriate levels with no apparent cause and can be divided into two main types: receptive–expressive (with a predominant receptive component) and expressive. At the heart of receptive DLD is disordered auditory perception [2].

The typical clinical picture of DLD includes delayed speech-language development with specific disorders within the brain’s structures. The distinctive features of phonemes and disorders are in the sequential arrangement of syllables (transpositions and reductions) in the receptive type, and problems with grammar (word categories and syntax) and semantic and association language functions in expressive type. In some cases, problems with perception are so conspicuous that the child appears to have a hearing disorder, as they do not understand common conversation and elicit the impression of disorientation [3,4].

Disordered phonemic hearing is common (e.g., difficulties in distinguishing voiced from voiceless sounds), and many children have limitations in expressive phonology. The ability to remember phonological information is poor; there is a specific impairment of short-term memory specialized for learning vocabulary [5]. Children with DLD cannot interpret language even if they do not understand the words, and they do poorly on story comprehension tasks. Comprehension difficulties are due to their expressive limitations [6].

The disordered temporal processing of sound (speech) is assumed, as well as disorder in the integration of auditory and speech connections. This deficit and its relationship with maturation theory and disorders in stimuli processing is related to higher cognitive functions and is considered one of the possible etiologies of DLD [7,8].

Affected children are not able to interpret communication on a basic level or recognize key words essential for the content of communication. They suffer from short-term memory disorder that causes the imperfect fixation of speech patterns in speech development and complicates further education and speech rehabilitation. Phonemic hearing disorder and the imperfect differentiation of speech sounds lead to various forms of impaired expressive language abilities. Manifestations of listening difficulties are always part of a more general disorder (e.g., auditory processing disorder (APD)) [9,10,11].

DLD is frequently associated with other neurodevelopmental diseases, such as developmental coordination motor disorder. Children may reach motor milestones late [12] and may present symptoms of attention deficit hyperactivity disorder (ADHD) [13]. However, the associated difficulties are not restricted to only the early developmental period. DLD often leads to dyslexia and may have a negative impact on the child’s self-esteem and well-being. In some children, this may lead to restricted social, academic, and occupational activities even beyond adolescence and into adulthood [1].

At present, the specific etiological factors leading to DLD are not known. Genetic predisposition has been assumed for many years [14] and is supported by findings of various genes that could contribute, as suggested by Li and Bartlett [15], to phonological short-term memory. At a neural level, perisylvian brain areas that contribute to language processing are often affected [16]. However, the exact mechanisms leading to these neural abnormalities are not known. Additionally, the range of cognitive or behavioral difficulties associated with DLD is not well understood.

Isolated epileptiform activity (IEA) appears to influence cognitive development [17,18], and there is clear evidence that IEA during sleep in particular affects language [19]. Epileptiform discharges in wake and sleep electroencephalograms (EEGs) and their causal connection with DLD have been studied for many decades. A meta-study by Systad et al. [20] reported that, in 55 studies including 2793 children, IEA was six times more prevalent in children with speech and language impairments than in children without developmental impairments. The overall pooled prevalence of IEA was 27.3% (ranging from 8.1% in speech impairment to 25.8% in language impairment and 51.5% in language regression). Sleep EEGs detected a significantly higher prevalence of epileptiform discharges than the wake EEGs. Studies that have conducted wake EEGs found IEA in 18.8% of cases, while studies conducting EEGs during sleep detected IEA in 37.1% of cases. Epileptiform discharges were detected more frequently during night sleep (58.1%) than in daytime sleep (26.7%). The prevalence of IEA was influenced by the presence of epilepsy and the age of the children, where children showed a higher incidence of IEA.

The aim of our retrospective study of DLD children was to characterize DLD subgroups and their phonemic hearing and to analyze their association with perinatal and genetic risk factors, neurodevelopmental comorbidities, and the results of wake EEGs and nocturnal video EEGs or polysomnography (PSG) records.

## 2. Subjects and Methods

Fifty-five children underwent diagnostic assessment for DLD at our department of phoniatrics and subsequently underwent neurological, EEG, and PSG examinations at our neurology department. All children also underwent psychological tests to estimate discrepancies between their verbal and nonverbal intellects. The children´s parents provided signed, informed consent to participate in the study, which was approved by the Ethics Committee of the General University Hospital in Prague and conducted in accordance with the Declaration of Helsinki (1427/08 S-IV (individual research), 16 October 2008).

### 2.1. Phoniatric Examination

All children were audiologically normal, according to pure tone audiometry and tympanometry. The children were evaluated by the widest spectrum of examination methods available. Detailed results (analysis of language levels and speech functions, phonological awareness, speech audiometry, and the Token test for children) facilitated the comparison of two different groups of children with DLD: receptive–expressive and expressive.

All children were examined by the Czech test of phonemic hearing [21] that provides information about phoneme identification skills. The child does not need to speak, but instead taps or clicks on a picture of the recognized spoken word (with phoneme opposite). The results were evaluated as percentages of correct answers. The aim was to analyze the role of phonemic hearing in children suffering from receptive and expressive forms of DLD.

The requirements for testing included normal peripheral hearing (pure tone audiometry) and sufficient receptive language skills (basic vocabulary and knowledge of one- or two-syllable words).

### 2.2. Psychological Tests

All children were examined by a childhood psychologist. Raven tests for small children and the Wechsler Preschool and Primary Scale of Intelligence for Children were administered, depending on the age of the child. Visual memory skills were measured as well, and handedness was classified. All diagnostic methods have been described previously in [22]. The diagnosis of ADHD was based on the Diagnostic and Statistical Manual for Mental Disorders, 4th ed (DSM-IV) classification [23].

### 2.3. Neurological Examination

All children, together with their parents, underwent face-to-face interviews focused on personal and family history, and detailed neurological examinations were also performed. Attention was particularly focused on perinatal risk factors, reaching motor milestones, coordination of motor abilities, history of epilepsy, and deficit of attention or hyperactivity. Specific learning disabilities could not be evaluated due to the young ages of the children. If a child presented mild disseminating neurological soft signs, neuroimaging methods were performed.

### 2.4. EEG and Nocturnal Video EEG or PSG Study

A wake EEG was performed by standard methods (duration of 20 min) and included hyperventilation and photic stimulation in all 55 children. Recordings were evaluated as normal (with respect to age) or abnormal. Abnormalities were classified as (1) non-epileptiform (usually slowing background activity or diffusing slow waves) or (2) epileptiform. The presence of spikes (transient, clearly distinguishable from background activity, and lasting 20–70 ms) and sharp waves (same as the spikes, but lasting 70–200 ms), either alone or accompanied by slow waves (being of higher amplitudes than the spikes or sharp waves), isolated or in bursts, were considered as epileptiform activity. Attention was focused on the localization of focal epileptiform changes and their lateralities.

An overnight EEG examination was done for all 55 children. In 21 children, nocturnal video EEG monitoring was performed, while 34 children underwent overnight video PSG examinations with extended 10–20 EEG montages [24]. The presence of epileptiform discharges was evaluated as described for wake EEGs.

### 2.5. Statistical Analyses

Statistical analyses were performed using Microsoft Excel 2010 and MedCalc software. The chi-square test in frequency tables, or Fisher´s exact test in the case of expected values ˂5, was assessed. McNemar’s test for related measures in 2 × 2 tables was also used. A two-group t-test for comparison of two means, or the Mann–Whitney test for testing subgroups ˂15 in size, was chosen, and *p*-values less than 0.05 were considered as statistically significant.

## 3. Results

### 3.1. Clinical Data

**Figure 1:**
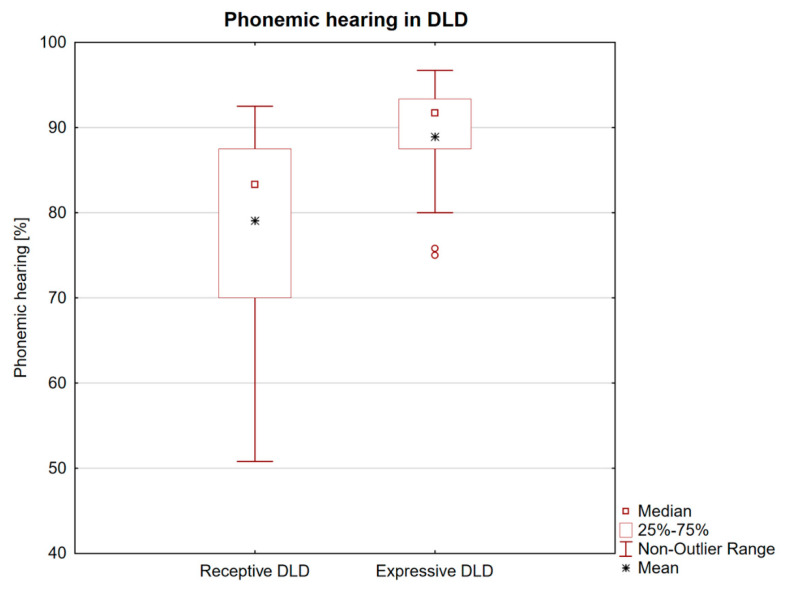
Comparison of phonemic hearing (Czech test of auditory discrimination) in developmental language disorder subtypes (receptive and expressive). The box plot shows that children with the receptive form showed a significantly increased number of phonemic discrimination errors. Two expressive DLD children represent outlying values.

Clinical data are summarized in Table 1. Fifty-five DLD children (42 male, mean age 6.2 ± 1.4 years, range 4–9 years) were included in the study. Right-handedness prevailed in 81.8% of cases (45 subjects). The receptive form of DLD was classified in 34 children (63%), while the expressive form was found less frequently in 20 children (37%). Poor cooperation in one child did not permit exact classification. Phonemic hearing in the whole group varied between 50.8–97.5%, with a mean value of 83.1% ± 10.6. Children with the receptive form of DLD had a significantly lower mean value of phonemic hearing (79.1% ± 10.9) than those with the expressive form (89.7% ± 6.2) with *p* < 0.001. Figure 1 shows a comparison of phonemic hearing severity in the receptive and expressive forms of DLD.

A positive family history of specific language impairments was found in 6 children (11.1%), and unspecified speech disorders in close relatives were found in 11 further families (20.4%). No differences with respect to receptive and expressive forms were found. Surprisingly, prenatal or perinatal risk factors (e.g., threatened abortion, preterm delivery, caesarean section due to fetal indication, and neonatal asphyxia) were present in both DLD groups (*n* = 19, 34.5%), and if neonatal icterus was considered a further risk factor (*n* = 9, 16.4%), the amount of affected children increased to one half of the whole cohort.

A high percentage of developmental comorbid disorders were also found. Developmental motor coordination disorder was diagnosed according to physician records in 33 children (61.1%); in older children, this was frequently in combination with ADHD manifestations. ADHD was the most frequent comorbidity and was diagnosed in 39 children (70.9%). Specific learning disabilities, particularly dyslexia, could not be evaluated due to the young age of the cohort. The Wechsler Scale of Intelligence for children showed subnormal IQ values (70–80) in 5 cases (9.1%). Nine children (16.4%) had histories of clinical absences.

Neuroimaging methods were performed in 32 cases, 26 of which were negative. In only six of the examined children, a mild, non-specific focal abnormality was found in different white matter areas, and in one patient, cavum septi pellucidi was found.

### 3.2. Wake EEG and Nocturnal EEG and PSG Findings

**Figure 2:**
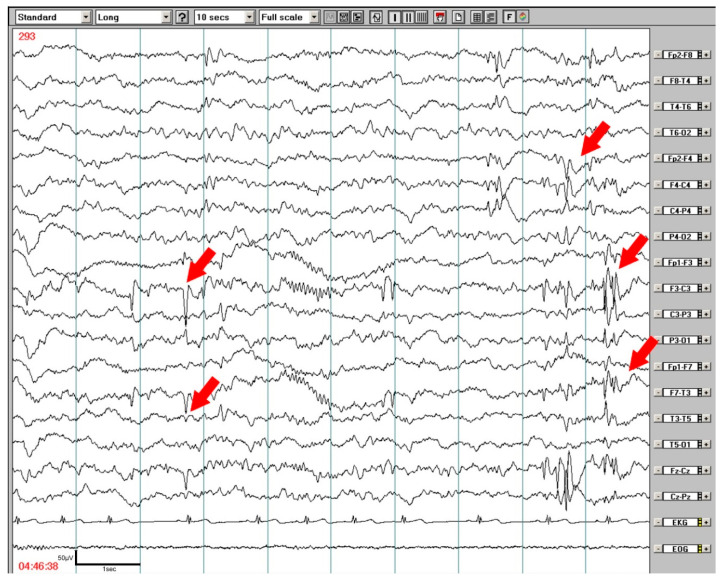
An 8-year-old boy with developmental language disorder. Nocturnal EEG monitoring–NREM (non-rapid eye movement) sleep, stage 2. Focal epileptiform discharges predominated in the left fronto-temporo-central areas.

Almost one half of the DLD children (49.1%) showed abnormalities on wake EEG with slowing background activity or diffuse slow waves that did not correspond to the child´s age. Epileptiform discharges on wake EEGs were predominantly focal, and frequently occurred bilaterally, predominantly in temporal or temporo-parieto-occipital regions (77.5% of recordings), and less frequently in fronto-central and rolandic areas. In sleep recordings, the focal activity shifted from posterior areas to fronto-temporo-central areas (89.5% of recordings; *p* < 0.001), only rarely appearing in parieto-occipital regions. Generalized discharges also increased during sleep. These findings are summarized in Table 2. Daytime wake EEG showed epileptiform discharges in 20 children (36.4%), in nocturnal EEG/PSG recordings epileptiform abnormalities were found in 30 children (55.6%). The amount of focal as well as generalized epileptiform discharges were enhanced during sleep; focal abnormalities were generally bilateral on wake as well as sleep recordings, where left predominance was enhanced. An exampleof EEG abnormalities during NREM (Non-Rapid Eye Movement) sleep is shown in Figure 2. The majority of epileptiform discharges appeared in NREM sleep, only 3 children presented focal epileptiform discharge in REM (Rapid Eye Movement) sleep.

However, we did not find any dependence of epileptiform discharges on wake EEG or nocturnal EEG/PSG recordings with perinatal risk factors, genetic predisposition, handedness, nor on developmental comorbidities (developmental motor coordination disorder, ADHD). This is likely due to the relatively low number of children followed, no increase of epileptiform abnormality was found in children with clinically-manifest epilepsy. Also, no relation to handedness or disseminating neuroimaging abnormalities was seen.

We also did not find any dependence of phonemic hearing on epileptiform abnormalities on wake EEG as well as nocturnal EEG/PSG recordings. Table 3 shows epileptiform findings in receptive and expressive forms of DLD. Despite a greater amount of almost all epileptiform abnormalities in the receptive form of DLD, no statistical differences were found during wake or sleep EEGs between these two groups. Even when dividing receptive and expressive DLD children in two cohorts according to disease severity, no statistical differences were found.

## 4. Discussion

A great deal has been written on how children learn to speak, but the development of language comprehension has been a relatively neglected topic. Comprehension is not a unitary skill. To understand spoken language, one needs the ability to classify incoming speech sounds, to relate them to a mental lexicon, to interpret the propositions encoded by word order and grammatical inflections, and to use information from the social context to select the one used by the speaker [25]. Children with more selective phonological impairments, and with difficulties in tasks that involve phoneme identification (e.g., our subjects with predominant receptive DLD), also had poor results in phonemic hearing. Their poor phoneme perception reflects a much more fundamental problem in discriminating a wide range of auditory stimuli, both nonverbal and verbal. Their expressive disturbances are a manifestation of their intake or decoding impairments. Language development depends in part on the accurate perception of the auditory input comprising speech, and children with reading disorders and language impairments may both be characterized by difficulties in low-level auditory processing skills [26].

Minor neurodevelopmental abnormalities, genetically transmitted predisposition, and acquired risk factors during the prenatal or perinatal period may create vulnerability toward DLD. A specific genetic predisposition to DLD was found in 11% of our subjects, and a family history of unspecified speech disorders in close relatives was found in a further 20%. Positive prenatal or perinatal risk was even higher; 51% of children had histories of threatened abortions, antepartum hemorrhages, preterm deliveries, caesarean sections due to prolonged delivery, or neonatal asphyxia or icterus. Complicated pregnancies or deliveries that may cause developmental or hypoxic brain injuries with neuronal damage may predispose children to all neurodevelopmental disorders [27]. Autism spectrum disorder is one of the best-studied diseases; according to Keller et al. [28], almost one half (43.3%) of persons with autism have a history of prenatal, perinatal, and postnatal risk factors. Additionally, developmental coordination disorder and ADHD, common comorbidities with DLD, are frequently associated with perinatal risk factors. In our DLD cohort, 61% of children showed developmental coordination disorder, and a greater number showed ADHD (71%).

The connection between ADHD and language impairment has been studied for many years [13,29,30]. Children with ADHD generally have worse language parameters and, according to some authors [29], also have more frequent abnormal EEG findings with epileptiform discharges than children with specific language impairment alone.

A high number of comorbidities may, therefore, influence the results of wake EEGs and nocturnal EEG or PSG recordings. However, findings of epileptiform activity have been reported as the most common EEG abnormality in children with specific language impairment since the 1990s [31,32,33], and they were related by some authors to architectural dysplasia and neuronal migration disturbances in affected children [34]. Focal epileptic discharges were thought to cause cognitive impairments [35,36,37] and language deficits [38]. It seems evident that language impairment is affected particularly by epileptic discharges during sleep, due to the disruption of consolidation processes and memory [19,39]. A maximum of focal epileptic discharges is usually localized in the temporal and rolandic regions, more frequently on the left side, which has a specific role in language development [40].

Our wake and nocturnal EEG and PSG findings were in agreement with some previous reports. Wake EEG abnormalities were found in almost one half of our subjects, similar to Mehta et al. [41], and sleep recordings showed enhanced epileptiform discharges in both of our DLD groups, though more prominently in the receptive group. Focal discharges were frequently localized bilaterally, including in the temporal regions. In sleep recordings, the focal activity shifted to the fronto-temporo-central areas. Sleep spindles, generated by the thalamus during NREM sleep and spread throughout the neocortex, play an essential role in both sensory processing and long-term memory consolidation [42]. Spindles are localized mostly over frontal regions in children, while central or parieto-central areas prevail later in adolescence and adulthood.

Likely due to the relatively low number of DLD subjects, we did not find any dependence of epileptiform discharges on perinatal risk factors, genetic predisposition, or handedness, nor on developmental comorbidities (e.g., developmental motor coordination disorder and ADHD) or epilepsy.

At present, the well-known gene forkhead-box-protein P2 (FOXP2), which participates in structural development of the brain in DLD children, is active during embryonal evolution and plays a role in neuronal migration in the cerebral cortex and its circuits and connections to the basal ganglia, thalamus, and cerebellum [43,44]. Neuronal migration in different brain areas may evoke minute epileptogenic lesions (e.g., heterotopias, focal cortical dysplasia, and mild gliosis) that may be evident on a magnetic resonance imaging (MRI) and recorded by an EEG examination. However, not only structural, but also functional brain networks may take part in epileptiform changes and their localization.

Our study may be limited due to its retrospective character. Due to this study design, dichotic tests were only used in part of our subjects and, therefore, were not reported. At present, we are also not able to confirm if some of our DLD subjects on antiepileptic treatment will profit from this medication. Their language prognosis should be confirmed by future observation.

## 5. Conclusions

Our study confirms the frequent association of DLD with other developmental diseases. Motor coordination disorder was detected in 61.1% of the subjects and ADHD in 70.9%. Almost one half of the perinatal risk factors were found in receptive as well as expressive DLD forms. Receptive DLD children had significantly lower mean phonemic hearing than those with the expressive form. Wake EEG examinations showed epileptiform discharges in 36.4% of the whole DLD group, and nocturnal EEG and PSG recordings enhanced epileptiform discharges in up to 55.6% of the children. While the wake EEG examination showed focal discharges predominantly in the temporal or temporo-parieto-occipital regions bilaterally, focal activity was shifted to the fronto-temporo-central areas in sleep recordings. No significant differences were found between the receptive and expressive DLD forms. Our results confirm the importance of nocturnal recordings with the enhancement of epileptiform activity, which may play a role in functional language disintegration in DLD children.

## Figures and Tables

**Table 1:** Developmental language disorder (DLD) clinical data (*n* = 55).

Children (*n*/%)		Additional Notes
male	42/75	
age (years) 6.2 ± 1.4		
range 4–9 years		
right-handedness	45/81.8	
DLD forms (*n*/%)		
receptive	34/63.0	1 child was not precisely classified due to limited exact cooperation.
expressive	20/37.0	
Family predisposition (*n*/%)		Data missing in one family.
specific language impairment	6/11.1	
unspecified speech disorder	11/20.4	
Perinatal risk factors		
(including neonatal icterus)	28/50.9	
Developmental comorbidities		
DMC	33/61.1	Data missing in one child.
ADHD	39/70.9	
IQ 70–80	5/9.1	
Epilepsy (clinical absences)	9/16.4	

Legend: DMC = developmental motor coordination disorder; ADHD = attention deficit hyperactivity disorder; and IQ = intelligence quotient (Wechsler Scale).

**Table 2:** Epileptiform discharges: Wake EEG and overnight sleep EEG/PSG (*n* = 55).

Epileptiform Discharges	Wake EEG Number (%)	Nocturnal EEG/PSG Number (%)	*p*-Value
Total	20 (36.4)	30 (55.6)	0.0124
Focal left-sided	12 (21.8)	23 (41.8)	0.008
Focal right-sided	13 (23.6)	21 (38.2)	0.032
Generalized	4 (7.3)	10 (18.2)	0.058

Note: one child may have a combination of different epileptiform discharges (McNemar test).

**Table 3:** Epileptiform discharges: Wake EEG and overnight sleep EEG/PSG in receptive and expressive form of DLD (*n* = 54).

Epileptiform Discharges Wake EEG	Expressive DLD (*n* = 20) Number (%)	Receptive DLD (*n* = 34) Number (%)	*p*-Value
Total	6 (30)	14 (41)	NS (0.41)
Focal left-sided	3 (15.0)	9 (26.5)	NS
Focal right-sided	4 (20.0)	9 (26.5)	NS
Generalized	2 (10.0)	2 (5.9)	NS
**Epileptiform discharges** **Nocturnal sleep recordings**			
Total	9 (45)	21 (62)	NS (0.23)
Focal left-sided	7 (35.0)	16 (47.1)	NS
Focal right-sided	6 (30.0)	15 (44.1)	NS
Generalized	3 (15.0)	7 (20.6)	NS

Note: one child may have a combination of different epileptiform discharges (McNemar test).
